# An Improved Multiple Competitive Immuno-SERS Sensing Platform and Its Application in Rapid Field Chemical Toxin Screening

**DOI:** 10.3390/toxics10100605

**Published:** 2022-10-12

**Authors:** Jiefang Sun, Zixuan Wang, Ling Yang, Yi He, Rui Liu, Wei Ran, Zhanhui Wang, Bing Shao

**Affiliations:** 1 Beijing Key Laboratory of Diagnostic and Traceability Technologies for Food Poisoning, Beijing Center for Disease Prevention and Control, Beijing 100013, China; 2 Department of Food and Bioengineering, Beijing Vocational College of Agriculture, Beijing 102445, China; 3 School of National Defence Science&Technology, Southwest University of Science and Technology, Mianyang 621010, China; 4 State Key Laboratory of Environmental Chemistry and Ecotoxicology, Research Center for Eco-Environmental Sciences, Chinese Academy of Sciences, Beijing 100085, China; 5 College of Veterinary Medicine, China Agricultural University, Beijing 100193, China

**Keywords:** multiple immunoassay, SERS assay, rodenticides, non-fouling interface

## Abstract

Improving the signal-to-noise ratio (SNR) by amplifying the outputting signal or reducing nonspecific binding (NSB) are the key techniques in multiple immunoassay. Aiming at these issues, this paper presents an improved multiple indirect competitive immune surface-enhanced Raman scattering (ci-SERS) assay for the rapid screening of highly toxic rodenticides in food and biological samples, which ensured remarkable accuracy, ultra-sensitivity and reproducibility. The non-fouling polymer brush grafted magnetic beads (the MB@P-CyM) were prepared as multiple competitive recognition substrates after conjugating triplex haptens (the MB@P-CyM-hap). It was demonstrated that the particular 3D hair-like structures of P-CyM not only facilitate conjugate high-density hapten but reduce the steric hindrance from SERS probes recognition, thus enhancing SNB. On the other hand, Au nanoflowers (AuNFs) of high SERS activity were synthesized using a simple one-pot hydrazine reduction. For simultaneously detecting three highly toxic rodenticides, i.e., diphacinone (DPN), bromadiolone (BRD) and tetramine (TET), the obtained AuNFs were fabricated as a SERS-encoded nanoprobe cocktail after successively labeling mono-antibodies/Raman probes. By integrating the MB@P-CyM-hap with the SERS-encoded cocktail, a highly sensitive multiple SERS assay was achieved in less than 2 h with a limit of detection of 0.62 ng mL^−1^ for BRD, 0.42 ng mL^−1^ for TET and 1.37 ng mL^−1^ for DPN, respectively. The recoveries of these rodenticides in spiked food and biological samples were determined and ranged from 72 to 123%. Above all, the proposed modifications show remarkable improvements for high efficient multiple chemical toxin immunoassay.

## 1. Introduction

The simple, accurate and rapid screening multiple analytes of interest plays a critical role in early disease diagnosis, food safety supervision as well as environmental surveillance [1,2,3]. Considering the complexity of the real food and biological samples, the key issue for various immune-sensors is in improving the signal-to-noise ratio (SNR), either by amplifying outputting signal or reducing nonspecific binding (NSB), causing false results [4]. Simultaneously, simplifying testing procedures and improving testing speeds are also highly desired. Currently, various highly sensitive signal reporting modules are putting forth new ideas [5,6]. However, strategies to reduce nonspecific binding in the sensing processes are slightly insufficient, [7,8] due to lacking adequate and deep understanding on adsorption/desorption, energy exchange as well as electron transfer occurred on nano-interfaces. Surface-enhanced Raman spectroscopy (SERS) is considered as an ideal tool for multiplex detection owing to its technical simplicity, excellent sensitivity and superior encoding capability [9,10]. By synthesizing highly sensitive SERS encoders with anisotropic plasmonic nanostructures, SERS-based multiplex competitive immunoassays have been proposed [11,12,13,14]. However, the immunoassays are proposed in the native “noisy” environments of lower fouling, and improving reproducibility is still not completely realized [7,15,16].

Chemical toxins-induced food poisoning accounted for more than 80% of the total foodborne deaths in China [17]. Among all of the chemical toxins, rodenticides, represented by diphacinone (DPN) [18], bromadiolone (BRD) [19] and tetramine (TET) [20], were the most common toxics, which have caused thousands of accidental or intentional poisoning events [21]. As beneficial complements for instrument assay [22], field screening these rodenticides in one simple test could not only remarkably improve treating efficiency, but it is also in line with future demands for rapid, cheap and high-throughput field screening toxins.

**Scheme 1:**
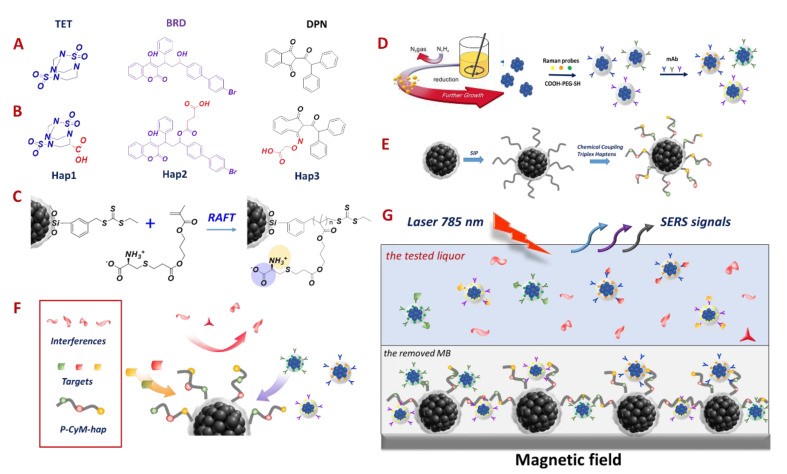
Chemical structures of (**A**) three rodenticides and (**B**) their corresponding haptens; (**C**) polymerization of the CyM on the MB based on the RAFT strategy; (**D**) synthesis of the AuNFs and their following label processes for constructing the specific SERS-encoding cocktail; (**E**) the preparation of the MB@P-CyM-hap; (**F**,**G**) the sensing principle was based on the multiple competitive immunoassay format: simultaneously mixing the mAb-labeled SERS encoding cocktail and the MB@P-CyM-hap with the rodenticide-spiked sample solutions. After reaching the recognition equilibrium, a magnet field was used to remove the MB@P-CyM-hap/SERS probes from the solutions, and the supernatant-containing SERS cocktails were collected for quantification.

Benefiting from their adjustable properties and flexible spatial structures, non-fouling polymer brushes offer attractive alternatives to engineer sensing interfaces for enhancing specific molecular recognition, rejecting nonspecific adsorption, amplifying the detecting signal as well as maintaining signal stability in complex working matrices [23,24]. In this work, an improved multiple competitive immuno-SERS assay was proposed for rapid screening DPN, BRD and TET (the chemical structures of these toxins are shown in Scheme 1A) in a single no-wash test. A non-fouling polymer brush grafted with magnetic beads (MBs) which were appended with triplex haptens for DPN, BRD, and TET (the chemical structures of these haptens are shown in Scheme 1B) was constructed as a multiple competitive substrate (the MB@P-CyM-hap). It was demonstrated that the hair-like P-CyM acted as the scaffold for conjugating haptens and could remarkably enhance its specific capture capacity for SERS probes by relieving steric hindrance as well as reducing nonspecific adsorption. For enhancing sensitivity, Au nanoflowers (AuNFs) were prepared using a one-step hydrazine reduction in aqueous solutions. The obtained AuNFs were recognized as an outstanding SERS substrate featuring high activity, easy preparation and good reproducibility. After successively labeling with the mono-antibodies (mAb)/Raman probe pairs, a specific SERS probe cocktail for sensing DPN, BRD and TET was constructed. By combining the MB@P-CyM-hap with the SERS sensing cocktail, a rapid and accurate rodenticide screening in a wide range of real samples was achieved. Under the optimized condition, if there are no toxins in the system, all of the SERS probes would bind with the hapten-modified MB and then remove them from the system. Thus, the supernatant shows no SERS signal. On the contrary, when toxins are present in the system, they would bind with the SERS probes preferentially, thus blocking the SERS probe from binding with the hapten modified-MB, which makes the supernatant show a distinct SERS signal (as shown in Scheme 1F,G).

## 2. Material and Methods

### 2.1. Chemical and Instruments

Hydrogen tetrachloroaurate trihydrate (HAuCl_4_), 2,6-dimethylphenyl isocyanide (DMPI), 4-thiosalicylic acid (TSA), 5,5-dithiobis(2-nitrobenzoic acid) (DTNB), hydrazine hydrate (N_2_H_4_), thioctic acid (TA), and carbonyl-PEG-thiol (1.0 kD) were purchased from Sigma-Aldrich (St. Louis, MO, USA). The standard substances including TET, BRD, DPN, brodifacoum (BTF), difethialone (DFT), coumatetralyl (CMT), difenacoum (DFC), coumafuryl (CMF), diphacinone (DPC), and chlorophacinone (CPC) were purchased from J&K Chemical Technology (Beijing, China). Both the monoclonal antibodies 4G5 to DNP and the monoclonal antibody to BRD 15C1 was previously prepared, and 1G6 to TET were produced by our group and will be described elsewhere. Other chemical reagents were purchased from Beijing Chemical Co. Ltd., Beijing, China. Ultrapure water (resistivity of 18.2 MΩ cm) was obtained by a Milli-Q water purification system.

The extinction spectrum was recorded on a Shimadzu UV 3600 UV-vis-NIR spectrophotometer. The morphologies of various nanostructures were observed on a JEM-2100F transmission electron microscope (JEOL Ltd., Tokyo, Japan). A physical property measurement device (Cryogenic, 12 T Magnet) was used to characterize the magnetic properties of the MBs. Thermogravimetric analysis (TGA) was performed with a TGA Instruments device (Model TGA Q500) from room temperature to 800 °C at a heating rate of 10 °C per min under nitrogen gas (40 mL per min). An attenuated total reflection-Fourier transform infrared spectroscopy (ATR-FTIR, INVENIO Bruker) was used to characterize the MBs. The concentration of the AuNFs and the MBs was measured on an ICP-MS (Agilent 8800) and an ICP-OES (IRIS Advantage, Thermo Scientific, St. Louis, MO, USA), respectively.

### 2.2. Preparation of the MB@P-CyM-hap as Multiple Competitive Substrate

The synthesis of SiO_2_-capped Fe_3_O_4_ magnetic beads (MBs) was conducted referring to the previously reported method [25]. The polymer monomer, named cysteine methacrylate (CyM), was synthesized according to the reported methods [26]. The non-fouling polymer brush-grafted MBs (the MB@P-CyM) were synthesized via a typical RAFT process [27]. For grafting the CyM polymer brushes from the MBs, CTA-MBs (0.1 g), AIBN (20 mg) and the CysMA monomer (0.1 g) were dissolved in 10 mL of degassed water/methanol (1:1) solution. After N_2_ bubbling for 30 min, the system was sealed and heated under 80 °C. After reaction for 5 h, the MB@P-CyM were collected and then washed by DMF 3 times (as shown in Scheme 1C).

### 2.3. Chemical Conjugation of Triple Haptens on the MB@P-CyM

The non-fouling MB@P-CyM was chemically conjugated with triple hapten (as shown in Scheme 1E) via the EDC/NHS mediated amino-carboxyl coupling reaction. The triple haptens were simultaneously linked on the MB@P-CyM with the feed molar ratio of 1:1:1. The triple hapten mixture with a total amount of 1 mmol L^−1^ was firstly activated by NHS (10 mg mL^−1^) and EDC (20 mg mL^−1^) in 1.0 mL methanol. After reacting for 60 min, the MB@P-CyM (0.1 g in 5 mL methanol) were added in the solution for conjugation. The reaction was processed for 10 h under room temperature, and then, the liquid supernatant was removed. The obtained MB@P-CyM-hap was washed with acetone repeatedly and finally dispersed in the PBS buffer for further use.

For achieving the competitive substrate with different hapten density, three groups of the hapten mixtures (10, 5, 1 mmol L^−1^ in 1 mL methanol) with the mole ratio of 1:1:1 were linked on the MB@P-CyM to fabricate high (the MB@P-CyM-hap1), middle (the MB@P-CyM-hap2), and low (the MB@P-CyM-hap3) densities, respectively, and then, we tested their binding capacity with the specific SERS encoders.

Moreover, the amino-derived MBs was also prepared for modifying triple haptens (the MB@hap), and we examined the specific binding capacity. Both the MB@P-CyM-hap and the MB-hap were prepared to share the same Fe concentrations as determined by ICP-OES.

### 2.4. Constructing the mAb-Labeled SERS Probe Cocktail

The AuNFs were prepared using hydrazine reduction in aqueous phase at room temperature for the first time, which was regarded as a seedless and surfactant-free method (as shown in Scheme 1D). In detail, 2.0 mL of 50.0 mmoL^−1^ HAuCl_4_ was mixed with 0.5 mL of 10.0 mmoL^−1^ TA ethanol solution in 100 mL of D.I. water. After stirring for 5 min, 0.5 mL of the freshly N_2_H_4_ aqueous solution was rapidly injected to the solution. The color of the solution changed to blue immediately, indicating the formation of the AuNFs. After standing for 2 h at room temperature, the AuNFs were purified by ultra-centrifuging (8000 rpm for 5 min) and washed twice with water, finally dispersing in 10 mL D.I. water.

The achieved AuNFs were capped by the carbonyl-PEG-thiol and then successfully modified mAb and Raman probes, i.e., the AuNF@PEG/TSA-mAb_BRD_, the AuNF@PEG/DTNB-mAb_TET_ and the AuNF@PEG/DMPI-mAb_DPN_. In detail, the purified AuNFs were incubated with the carbonyl-PEG-thiol (1.0 µmoL^−1^) for 30 min. After centrifugation, the carbonyl-PEG capped AuNFs were reacted with the EDC/sulfo-NHS regents. After 30 min, 200 μL of 0.1 mg mL^−1^ mono-antibody solutions for DPN (mAb_DPN_), BRD (mAb_BRD_) and TET (mAb_TET_) were added, respectively, and then maintained overnight under 4 ℃ for conjugation. Finally, the mAb-labeled AuNFs (the AuNF@PEG/mAb_BRD_, the AuNF@PEG/mAb_TET_ and the AuNF@PEG/ mAb_DPN_) were centrifuged at 8000 rpm for 5 min to remove unbound antibodies and washed with the PBS buffer. Subsequently, 10 μL of TSA (1.0 mmoL^−1^), DTNB (1.0 mmoL^−1^) and DMPI (1.0 mmoL^−1^) were added into 1.0 mL of the purified mAb-AuNF solutions, respectively, thus obtaining SERS encoders, i.e., the AuNF@PEG/TSA-mAb_BRD_, the AuNF@PEG/DTNB-mAb_TET_ and the AuNF@PEG/DMPI-mAb_DPN_, which were finally re-suspended into 300 μL of 0.1% BSA solutions and stored under 4 °C before use. The concentrations of these SERS encoders were prepared to the same Au contents (0.5 mmoL^−1^) as measured by ICP-MS after the aqua regia digestion.

### 2.5. Detection of DPN, BRD and TET in Buffer

For examining the sensing performances, 1.0–10.0 μL of the standard toxins was added to the MB@P-CyM-hap or the MB-hap solutions (50 µL), and then, their corresponding SERS probe cocktails were added. After 30 min, the free SERS probe solutions were collected after magnetic separation for Raman interrogation. The results were analyzed by linear regression and used to verify the accuracy of the immunoassay. The selectivity of the assay was examined by using chemical analogue (0.5 µg mL^−1^), and the subsequent SERS measurements were processed consistently with the above procedures. The detailed sample preparation and measurements are given in the Supplementary Materials.

### 2.6. Screening DPN, BRD and TET in Spiked Biological and Food Matrices

The SERS-encoded cocktail (100 μL) was prepared by mixing the AuNF@PEG/TSA-mAb_BRD_, the AuNF@PEG/DTNB-mAb_TET_ and the AuNF@PEG/DMPI-mAb_DPN_ with the final molar ratio of 2:1:3, and then, the MB@P-CyM-hap solutions (50 µL) were added in EP tubes separately. The negative human serum and urine samples were supplied by the Beijing Center for Disease Prevention and Control (Beijing, China). The negative food samples including milk and chicken were purchased locally. For method validation, different concentrations of BRD, DPN and TET standards were spiked into the real samples to the final concentrations of 5.0 ng mL^−1^ and 10 ng mL^−1^, respectively. Briefly, 1.0 mL or 1 g of samples was extracted with ethyl acetate (1.0 mL) on the vortex for 5 min, and the mixture was centrifuged for 5 min at 3500× *g*. After being dried using nitrogen flow, the dried residue was reconstituted with PBS for measurement.

### 2.7. SERS Measurement

A Raman spectrometer (inVia Renishaw, UK) was utilized for SERS measurements. The mAb-labeled SERS probe cocktail was dispersed on quartz cells and then measured under the laser excitation wavelength of 785 nm. The laser output was performed by a 50 L × 0.3 NA air objective lens. For each measurement, the laser power and exposure time were set at 10 mW and 5 s exposure with 10 accumulation, respectively.

## 3. Results and Discussion

### 3.1. Characterizations of the Non-Fouling MB@P-CyM-hap

**Figure 1:**
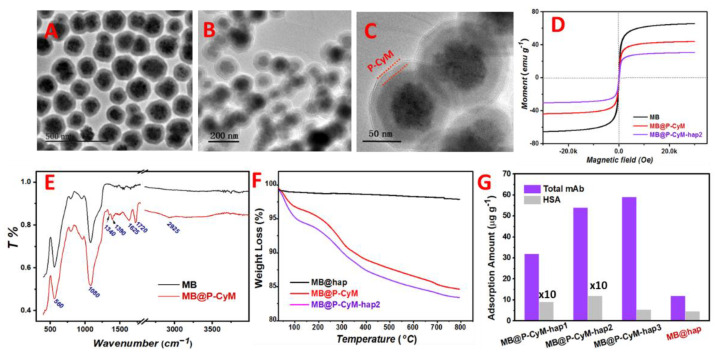
TEM images of the MB (**A**), the MB@P-CyM (**B**) and the zoomed-in image of the MB@P-CyM (**C**); (**D**) FT-IR spectra and (**E**) TGA curves as well as (**F**) the magnetization measurements of different modified MBs; (**G**) Evaluation of the binding and the non-specific adsorption characters for different hapten-modified MBs.

Surface geometries and chemical compositions of nanostructures have close relations with affinity recognitions [5]. In-depth research on the properties of the sensing interface are vital for constructing nanosensors [6,7]. Different from small molecule probes, SERS nanoprobes often possess tens of nanometers, the effect of steric hindrances on recognition and binding should be considered. The P-CyM is verified as an attractive non-fouling interface which contains abundant active sites for conjugating hapten. In this work, the P-CyM brushes were grafted from the MBs using a typical RAFT strategy. As shown in Figure 1A–C, the MB@P-CyM shows good dispersion and uniform morphologies with the inner core of ≈110 nm in diameter, the middle SiO_2_ protecting layer of ≈15 nm and the outer P-CyM brushes of ≈20 nm. The hydrophilic P-CyM made the MB@P-CyM have good stability in aqueous solution. Moreover, the saturation magnetization of the MB@P-CyM is measured as 30.5 emu g^−1^. The magnetization curves exhibit symmetry and passed through the origin, which ensures a facile separation and reusability of the MB@P-CyM from sample matrices (Figure 1D). FT-IR spectra (Figure 1E) of the MB@P-CyM are collected and compared with their precursors. The strong peaks around 1080 cm^−1^ assigned to Si-O are observed both in the MBs and the MB@P-CyM. Specifically, the stretching vibration of −CH_2_−, −COOH, −amino and the symmetric stretching vibration of C-O associated with P-CyM ligand appear at 2950 cm^−1^, 1720 cm^−1^, 1340 cm^−1^ and 1625 cm^−1^, respectively in the MB@P-CyM spectra. According to TGA analysis, a distinct mass loss of ≈14% is observed in the MB@P-CyM, which is higher than that of the NH_2_-MBs of ≈2% weight loss (Figure 1F).

The MB@P-CyM comprise abundant primary amino groups which enable convenient conjugation with triplex haptens with adjustable density. Different amounts of the hapten mixtures of the mole ratio of 1:1:1 were covalently linked on the MB@P-CyM with low (the MB@P-CyM-hap1), middle (the MB@P-CyM-hap2) and high (the MB@P-CyM-hap3) densities. As a control, the amino-MBs without grafting P-CyM were also conjugated with triple haptens (the MB@hap). For comparing the binding capacities of different modified MB, their concentrations were set as the same levels (in terms of Fe ions). Their specific bindings with the total mAb mixture as well as the nonspecific protein (HSA) were examined. It is found that the adsorption capacities of hapten-modified MB@ P-CyM for the triplet mAb mixture improved with the increase in the hapten density (Figure 1G). Under physiological condition, the MB@P-CyM-hap2 and the MB@P-CyM-hap3 show comparable binding contents for the total mAb, demonstrating the steric hindrance restrict saturating absorption. For all of these, the MB@P-CyM-hap show a better adsorption capacity than the MB@hap. The anti-fouling performance derived from the P-CyM shows degradation due to the hapten conjugation-induced polarity changes, as it is reflected that both the MB@P-CyM-hap1 and the MB@P-CyM-hap2 exhibit strong resistance to biofouling, with the nonspecific protein absorption as low as 1.2 μg g^−1^, but the value increases to 5.4 μg g^−1^ for the MB@P-CyM-hap3 (Figure 1G). The MB@hap without grafting the P-CyM exhibits a similar unspecific fouling to that of the MB@P-CyM-hap3. It is found that the MB@P-CyM-hap2 reaches a saturated adsorption within 30 min, suggesting that the flexible hair-like interface of the MB@P-CyM-hap2 facilitated the synergy affinity by reducing steric hindrance. As shown in Figure 1G, the MB@P-CyM-hap2 shows weak interaction with the nonspecific protein (HSA). Moreover, after storing under RT for 6 months under the freeze-dried state, the MB@P-CyM-hap2 shows no obvious adsorption drop, indicating their long-term chemical stability in aqueous solution. All of these results emphasize the vital role of the P-CyM brushes as the recognition interface on the MB. Comprehensively considering these results, the MB@P-CyM-hap2 is selected for further SERS sensing applications.

### 3.2. Fabricating the Specific SERS-Encoding Probes and Examining Their Sensing Capacities

**Figure 2:**
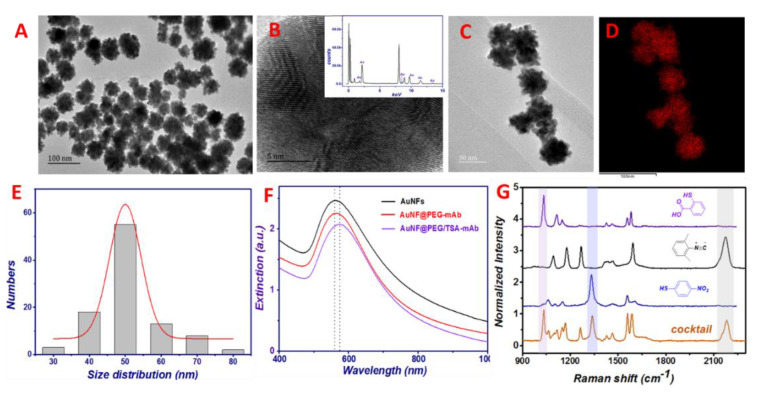
TEM images of the AuNFs (**A**) and the AuNF@PEG-mAb (**B**); the high-resolution AuNFs (**C**); zoomed-in TEM results of the AuNFs (**D**) and their corresponding EDS image; EDS result for the same area in Figure 2D (inner **B**); (**E**) the size distribution of the synthesized AuNFs; (**F**) the extinction spectra of the different modified AuNFs; (**G**) SERS signals of different modified AuNFs and the label SERS cocktail.

Both experimental and theoretical research have indicated that shapes can determine the properties of particular nanostructures such as sizes. In this paper, uniform AuNFs were synthesized by one-step reduction in an aqueous phase under room temperature. The formation mechanism of the AuNFs is depicted in Scheme 1D. The oxidation product of N_2_H_4_, i.e., N_2_, was generated in aqueous solution, interfering with the growth of Au seeds; thus, the flower-like Au nanostructure is obtained. The AuNFs were completely formed within 30 s and stayed the same afterwards. As shown in the TEM images (Figure 2A), the Au NFs are monodispersed with size distribution between 40 and 70 nm (Figure 2E). EDS results evidence the sole Au element component of these nanoparticles (Figure 2B,D). The HR-TEM image of the AuNFs is also given in Figure 2B, where the two lattice fringes with the d-spacing of 0.236 and 0.204 nm corresponding to the (111) and (200) planar distances of Au are shown. As shown in Figure 2F, the extinction spectrum of the AuNFs exhibits a distinctive surface plasma resonance (SPR) band at around 570 nm and showed blue color. It indicated that the AuNFs can remarkably enhance the Raman signal of probes (TSA in this research) by having a large surface-to-volume ratio, surface roughness as well as a plethora of nanogaps, which is more sensitive than that of the spherical AuNP of similar diameters. Statistically, the SERS enhancement of different batches of the AuNFs shows a relative standard deviation of 6.7%, indicating that the achieved AuNFs have well reproducible SERS performance.

In the SERS barcoding system, the AuNF@PEG was functionalized with different pairs of mAb and Raman reporters, i.e., the mAb_BRD_/TSA, the mAb_TET_/DTNB as well as the mAb_DPN_/DMPI, respectively. Comparing the original AuNFs with the SPR peak at 572 nm, it slightly shifts to 590 nm after successively labeling the Raman reporters and the mAb (Figure 2F). This result suggests that both encoding and ligand grafting procedures did not detriment the colloidal stability. For simultaneously screening multiple rodenticides, three typical Raman probes (MBA, DTNB, and DMPI) that generated unique Raman signals upon laser excitation were selected to encode SERS probes for BRD, TET and DPN, respectively. The SERS signals of each AuNF encoder and their mixtures are demonstrated in Figure 2G. It is found that three sensitive and non-interfering SERS peaks are clearly observed, which demonstrate that the cocktail of these SERS nanoprobes could serve as an effective tool for multiplex target screening. These specific SERS nanoprobes have similar negative charges around −24.1 ± 0.3 mV due to the presence of TA ligands, and the PEG ligand could effectively maintain the stability of them in the complex biological media, as it demonstrated that no obvious aggregation of the AuNFs is found in PBS or 10 times diluted human serum for over 1 month.

### 3.3. Sensing Performance of This Assay

**Figure 3:**
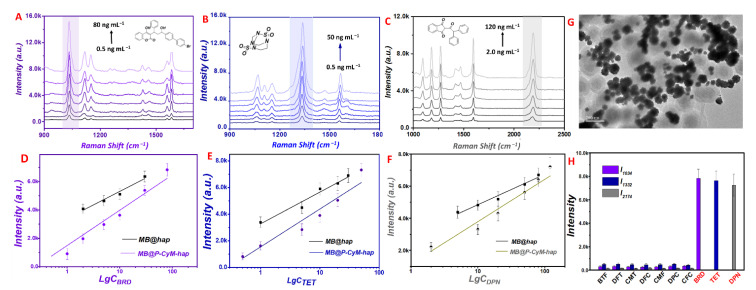
SERS spectra and the corresponding linear calibration curve for the detection of BRD (**A**,**D**), TET (**B**,**E**) as well as for DPN (**C**,**F**) using the multiple competitive MB with or without grafting P-CyM in PBS buffer. Each data point was the mean of triplicate analyses. (**G**) TEM images demonstrated the specific binding among the MB@P-CyM-hap and SERS nanoprobes. (**H**) Selectivity of this method. All of the interferences were at 1.0 μg mL^−1^.

For further verifying the advantages of this modified SERS platform, we examined the sensitivity and selectivity of the assay. As expected, by integrating the MB@P-CyM-hap2 with the SERS probes in this assay, gradually, SERS signal restorations as a function of the increasing concentration of targets are observed, as shown in Figure 3A–C. Similarly, SERS signal-enhancing trends are observed for TET, BRD and DPN, suggesting the potentials of quantitative analysis. Multiple calibration curves are plotted between peak intensities at 1034, 1332, and 2174 cm^−1^, i.e., the AuNF@PEG/TSA-mAb_BRD_ at 1034 cm^−1^ for BRD from 1.0 to 80 ng mL^−1^ (I_1034_ = 1896 × lgC_BRD_ + 3407) with a squared correlation coefficient (R^2^) of 0.962, and the AuNF@PEG/DTNB-mAb_TET_ at 1332 cm^−1^ for TET from 0.5 to 50 ng mL^−1^ (I_1332_ = 2367 × lgC_TET_ + 2573, R^2^ = 0.988) and the AuNF@PEG/DMPI-mAb_DPN_ at 2174 cm^−1^ for DPN from 2 to 120 ng mL^−1^ (I_2174_ = 2625 × lgC_DPN_ + 1154, R^2^ = 0.95) (Figure 3). In addition, limits of detection (LOD) were calculated as 0.62 ng mL^−1^ for BRD, 0.42 ng mL^−1^ for TET and 1.37 ng mL^−1^ for DPN based on three times the standard deviations of the control signals, which could meet the requirement of fast food poisoning screening. Moreover, TEM images also provide the evidence of forming the nanoassemblies (SERS nanoprobes binding on the surfaces of the MB@P-CyM-hap2) in the presence of different targets, which illustrates their successful binding (Figure 3G). The inter- and intra-day precisions are monitored as less than 16.24%. On the contrary, the sensing performance using the MB@hap under the same detecting conditions is not good as the MB@P-CyM-hap2 in the aspects of RSD, LOD and linear ranges, suggesting the outstanding advantages of using the non-fouling competitive interfaces. This results reveal that attributing to the particular 3D hair-like structures, the P-CyM interface not only facilitates conjugate high-density hapten but reduces the steric hindrance between the SERS probes, thus enhancing their recognitions and sensing capacity. Moreover, due to the specificity of the mAb and the introduction of the non-fouling sensing interface, the selectivity of the assay is satisfactory (Figure 3H).

### 3.4. Sample Analysis and Method Validation

Finally, SERS detection and quantification for BRD, TET and DPN in spiked biological and food samples were achieved in less than 2 h. The recoveries of the assay, as shown in Table 1, ranged from 72% to 123%, with CV values of 8.86 to 15.46%, indicating the good accuracy and precision of this method.

## 4. Conclusions

In this work, the improved multiple ci-SERS assay was proposed for the simultaneous rapid screening of TET, BRD and DPN in biological and food samples. The chemical improvements are demonstrated for achieving better sensing results: firstly, the non-fouling polymer brush was introduced on the magnetic colloid as the scaffold for conjugation triple hapten, thus achieving enhanced signal-to-noise ratios. Secondly, the uniform AuNFs of high SERS activity was prepared by a simple one-pot reduction, which ensures the well reproducibility and ultra-sensitivity of this assay. These results suggested that the established assay has advantages of sensitivity, rapid response, easy operation and reliability, and thus, it provides an excellent sensing platform in wide fields. Above all, this assay was anticipated as a user-friendly sensor for on-field toxins screening in wide environmental and food matrices.

## Figures and Tables

**Table 1:** Determination of TET, BRD and DPN in different spiked matrices using the proposed detecting strategy (data are mean value of 3 measurements).

Sample	Toxin/Spiked (ng mL^−1^)	This ci-SERS Assay	
		Detected (ng mL^−1^)	Recovery (%)
Human Serum	BRD/10.0	8.46	84.6
	TET/10.0	11.4	114
	DPN/10.0	7.32	73.2
Milk	BRD/10.0	7.78	77.8
	TET/10.0	11.8	118
	DPN/10.0	8.24	82.4
Chicken	BRD/10.0	7.21	72.1
	TET/10.0	11.8	118
	DPN/10.0	12.3	123

## Supplementary Materials

The following supporting information can be downloaded at: https://www.mdpi.com/article/10.3390/toxics10100605/s1, Figure S1: The synthesis route for the CysMA monomer; Figure S2: The synthesis route for the CTA; Figure S3: The synthesis route for the MB@P-CyM-hap; Figure S4: XRD results of different MBs; Figure S5: TEM images of the SERS encoding cocktail.
