# Supplementary material for: An Improved Multiple Competitive Immuno-SERS Sensing Platform and Its Application in Rapid Field Chemical Toxin Screening
Source: Toxics. 2022 Oct 12;10(10):605. doi: 10.3390/toxics10100605 (PMC9606884; doi:10.3390/toxics10100605)
Supplement: Supplementary file 1 [file toxics-10-00605-s001.zip › toxics-1907601-supplementary.pdf]

toxics

MDPI

An Improved Multiple Competitive Immuno-SERS Sensing Platform and Its Application in Rapid Field Chemical Toxin Screening

Jiefang Sun, Zixuan Wang, Ling Yang, Yi He, Rui Liu, Wei Ran, Zhanhui Wang and Bing Shao

1.1. Chemicals

L-cysteine, 3-(acryloyloxy)-2-hydroxypropyl methacrylate, anhydrous THF, 1-propanethiol, anhydrous acetone, CS_2, (4-(chloromethyl)phenyl)-trimethoxysilane dichloromethane, and 2,2'-azobisisobutyronitrile (AIBN) were purchased from J&K Chemical.

1.2. Preparation of food samples

The crude food and biological samples for this research were prepared from diluted human serum, orange juice, fresh milk, and sandwich. The solid samples were weighed and diluted with PBS buffer (1.0 mL per gram), then homogenized for 5 min, followed by centrifuging for 2 min at 1200 rpm to remove large residuals. The supernatant was frozen until used. The human serum was diluted 5 times with phosphate-buffered saline (PBS) buffer before analysis.

Briefly, 2.0 mL of plasma was extracted with ethyl acetate (10 mL) on the vortex for 5 min, and the mixture was centrifuged for 5 min at 3500 g. Next, 5 mL of the supernatant was separated and dried using nitrogen flow at 50 °C. Then, the dried residue was reconstituted using PBS buffer (2 mL, 0.01 mol L^{-1}, pH 7.4), and 50 μL of the pretreated sample was submitted to the sensing system.

For tissue samples, the total pretreatment time was approximately 1 h, because an extended period of sample drying under blowing nitrogen gas was needed according to the protocol using commercial ELISA kits. For liquid sample, it could be analyzed directly after dilution without centrifugation using this proposed method.

1.3. Synthesis of the cysteine methacrylate (CysMA) monomer

The CysMA was synthesized according to a previous reported method [1]. In detail, L-cysteine (15.13 g, 124.88 mmol) was dissolved in deionized water (100 mL), and then 3-(acryloyloxy)-2-hydroxypropyl methacrylate (29.43 g, 137.36 mmol) was added to this stirred aqueous cysteine solution. Subsequently, dimethylphenyl phosphine (20 μL, 147 μmol) was added to the aqueous reaction mixture and stirring was maintained for 2 h at 20 °C. The reaction solution was washed twice with ethyl acetate (50 mL) and dichloromethane (50 mL) repeatedly. Finally, the CysMA monomer product was isolated as a pure white solid (39.6 g, 94% yield) by freeze-drying the concentrated aqueous solution overnight.

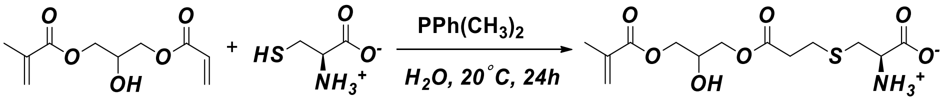

Figure S1. The synthesis route for the CysMA monomer.

1.4. Synthesis of the poly CysMA grafted MBs (the MB@P-CyM)

The reversible addition–fragmentation chain-transfer (RAFT) initiator, i.e., (propyl-4-(trimethoxysilyl)benzyl carbonotrithioate, CTA), was synthesized according to the reported methods [2]. In detail, 1-propanethiol (6.6 mmol) was charged into a stirred suspension of K_3PO_4 (1.02 g, 6.6 mmol) in anhydrous acetone (15 mL), followed by stirring for about half an hour. CS_2 (1.1 mL, 18 mmol) was added, and the solution turned to bright yellow. After stirring for another 10 min, (4-(chloromethyl)phenyl)-trimethoxysilane (1.43 mL, 6.6 mmol) was added, and the mixture was then stirred at ambient temperature in nitrogen atmosphere for 13 h. The mixture was concentrated, diluted with dichloromethane, and filtered off. After removing the solvent from the filtrate under reduced pressure, the resulting yellow residue was purified by column chromatography on silica gel using a petroleum ether/ethyl acetate gradient to yield a bright yellow oil.

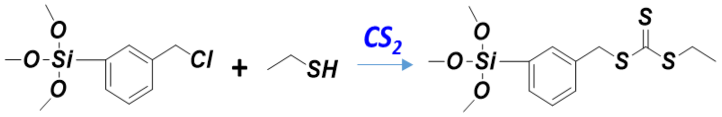

Figure S2. The synthesis route for the CTA.

The water-dispersible SiO_2-capped Fe_3O_4 microbeads (MBs) and the amino-modified MBs (NH_2-MBs) were prepared according to a previously reported method [3]. To modify the MBs with the RAFT initiator, 50 mg of CTA was added into 100 mL suspension of MBs in absolute ethanol (1.0 mg mL^{-1}), followed by refluxing under nitrogen for 5 h. The obtained CTA-MBs preparation was collected and washed with ethanol 3 times. Finally, the CTA-MBs preparation was suspended in ethanol for further use.

1.5. SERS measurement

A Raman spectrometer (inVia Renishaw, U.K.) was utilized for SERS measurements. The mAb-labeled SERS probe cocktail was dispersed on quartz cells, and then measured under the laser excitation wavelength of 785 nm. The laser output was performed by a 50 L× 0.3 NA air objective lens. For each measurement, the laser power and exposure time were set at 10 mW and 5 s exposure with 10 times accumulation.

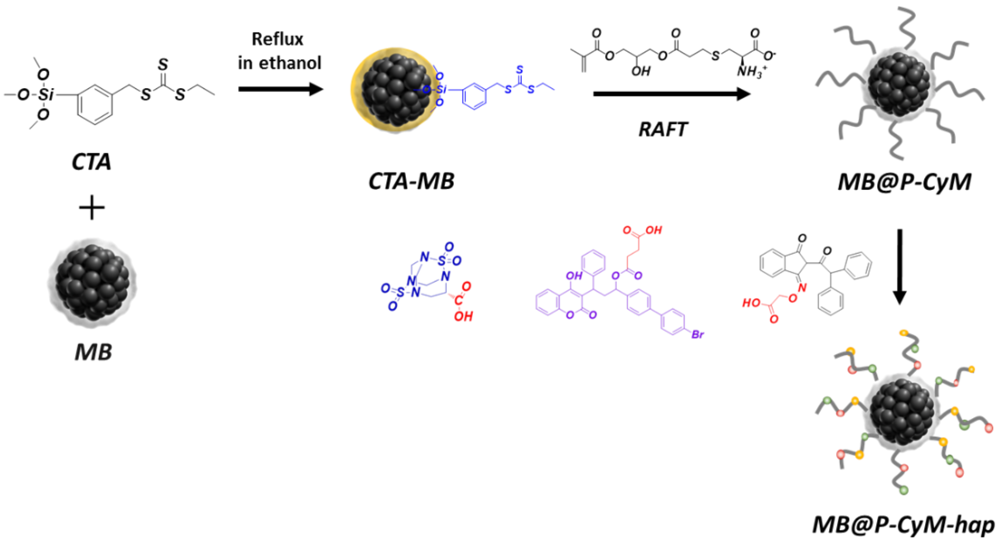

Figure S3. The synthesis route for the MB@P-CyM-hap.

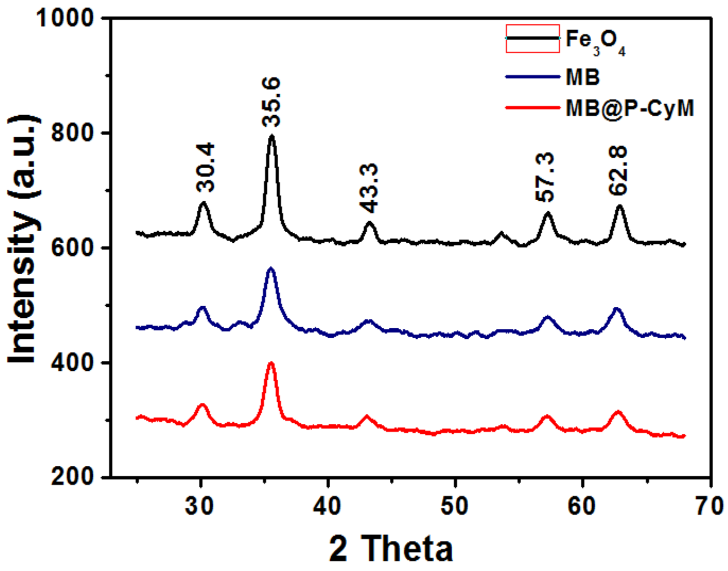

Figure S4. XRD results of different MBs.

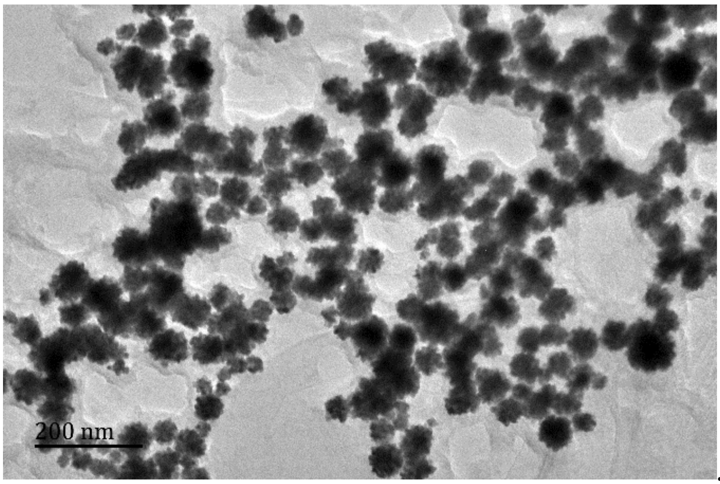

Figure S5. TEM images of the SERS encoding cocktail.

References

1. Alswieleh, A.M.; Cheng, N.; Canton, I.; Ustbas, B.; Xue, X.; Ladmiral, V.; Xia, S.; Ducker, R.E.; El Zubir, O.; Cartron, M.L.; et al. Zwitterionic Poly(amino acid methacrylate) Brushes. J. Am. Chem. Soc. 2014, 136, 9404–9413.
2. Qu, Z.; Hu, F.; Chen, K.; Duan, Z.; Gu, H.; Xu, H. A Facile Route to the Synthesis of Spherical Poly(Acrylic Acid) Brushes via RAFT Polymerization for High-Capacity Protein Immobilization. J. Colloid Interface Sci. 2013, 398, 82–87.
3. Deng, Y.; Qi, D.; Deng, C.; Zhang, X.; Zhao, D. Superparamagnetic High-Magnetization Microspheres with an Fe3O4@SiO2 Core and Perpendicularly Aligned Mesoporous SiO2 Shell for Removal of Microcystins. J. Am. Chem. Soc. 2008, 130, 28–29.
